# Patient Satisfaction With Single-Shot Spinal Analgesia for Labor: A Single-Center Study

**DOI:** 10.7759/cureus.83884

**Published:** 2025-05-11

**Authors:** Ivan Keser, Slavenka Straus, Denis Imamovic, Mirko Mihalj

**Affiliations:** 1 Department of Anesthesiology, General Hospital "Prim. Dr. Abdulah Nakaš", Sarajevo, BIH; 2 Clinic for Anesthesia and Reanimation, Clinical Center of the University of Sarajevo, Sarajevo, BIH; 3 Department of Anesthesiology, University Clinical Hospital Mostar, Mostar, BIH

**Keywords:** labor analgesia, low-resource settings, obstetric anesthesia, parturient satisfaction, single-shot spinal, spinal anesthesia, visual analog scale

## Abstract

Background

In low- and middle-income countries, particularly during the COVID-19 pandemic, the availability of epidural analgesia for labor has been limited due to a shortage of anesthesiologists. As an alternative, single-shot spinal analgesia can offer effective pain relief during childbirth.

Objective

The objective of the study is to evaluate patient satisfaction with single-shot spinal analgesia provided during labor.

Methods

This descriptive cross-sectional study was conducted at the General Hospital Sarajevo over a two-year period. Fifty parturients received single-shot spinal analgesia during the active phase of labor (cervical dilatation ≥ 5 cm). The analgesic mixture included 0.5% levobupivacaine (0.5 mL), fentanyl (25 mcg), and 0.9% NaCl (1 mL). Pain intensity was assessed using a Visual Analog Scale (VAS) before the procedure, 10 minutes afterward, one hour after administration, and during delivery. Data were collected using a structured questionnaire.

Results

The average duration of labor pain prior to the procedure was four hours. Mean VAS scores were 9 before the procedure, 3 after 10 minutes, 1 after one hour, and 4 during delivery. Spinal analgesia lasted approximately 110 minutes. During this period, 66% of parturients delivered, while in 34%, the effect of analgesia ended before delivery. All patients expressed satisfaction with the analgesia received.

Conclusion

Single-shot spinal analgesia provided effective pain relief and high patient satisfaction, even among those who did not deliver during the period of analgesic effect. It offered parturients sufficient time to rest and actively participate in the final stage of labor.

## Introduction

Labor pain is often described as one of the most intense pains a mother may experience in her lifetime, often compared to the pain experienced after a finger amputation. The stress caused by labor pain increases catecholamine levels, leading to an increase in cardiac output and peripheral vascular resistance, while simultaneously reducing uteroplacental perfusion. Additionally, intermittent pain caused by uterine contractions triggers episodes of hyperventilation. In the absence of supplemental oxygen, the compensatory hypoventilation periods between contractions may result in temporary episodes of hypoxemia in both the mother and fetus, which can have serious consequences for both [1].

Moreover, persistent and severe pain has been shown not only to cause physiological disturbances but also to have psychological consequences, such as postpartum depression, which can further compromise the mother's general and emotional well-being [2]. Therefore, relieving pain during labor is not only a humane act but a justified medical intervention that enables a safer, higher-quality, and less stressful delivery for both the mother and the baby. A mother’s request for pain relief is sufficient justification for providing analgesia during childbirth [3,4]. There is no scenario in which it would be acceptable for a person to endure severe, treatable pain while under medical care [5]. Epidural analgesia is a well-established technique considered the gold standard for pain relief during labor. It provides highly effective pain control, significantly contributing to maternal satisfaction [3].

Single-shot spinal analgesia, performed using traditional lumbar puncture techniques, has been shown to provide excellent pain relief with minimal side effects [6]. Its onset is faster than epidural analgesia, with many women experiencing effective pain relief within just 10 minutes [7]. However, this method has a limited duration, typically lasting between 60 and 120 minutes. If labor unexpectedly lasts longer, placement of an epidural catheter or repeat spinal injection may be required [8-10]. In low- and middle-income countries, due to a lack of trained anesthesiologists, especially during the COVID-19 pandemic, epidural analgesia is often not available to pregnant women. As a result, single-shot spinal analgesia is frequently used as an alternative.

In recognition of global disparities in labor pain management, the World Health Organization (WHO) recommends that all women should have access to effective pain relief during labor, adapted to local resources and individual preferences. In its 2018 intrapartum care guidelines, the WHO emphasizes offering evidence-based analgesic options, including regional techniques where feasible, and supports the exploration of alternatives in low-resource environments. This study aligns with those recommendations by evaluating single-shot spinal analgesia as a practical and effective method of labor pain relief in a setting where continuous epidural services are limited [11].

## Materials and methods

This descriptive cross-sectional study was conducted at the General Hospital "Prim. Dr. Abdulah Nakaš" in Sarajevo between 2020 and 2022. The study included 50 women who requested analgesia during labor. Informed consent was obtained from each participant who was offered the option of single-shot spinal analgesia.

The inclusion criteria encompassed healthy, term parturients aged 18-40 years, with singleton pregnancies in the active phase of labor (cervical dilation ≥ 5 cm), requesting analgesia, and without any known obstetric or anesthetic contraindications. The exclusion criteria included patient refusal, known coagulation disorders, therapeutic anticoagulation (low-molecular-weight or unfractionated heparin), localized infection at the puncture site, allergy to local anesthetics or opioids, neurological conditions, or suspected elevated intracranial pressure.

Spinal analgesia was administered following consent and under continuous hemodynamic monitoring. The procedure was performed with the patient in a seated position using a standard sterile technique at the L4/L5 intervertebral space, employing a 27-gauge pencil-point spinal needle.

The intrathecal solution consisted of the following components: 0.5 mL of 0.5% levobupivacaine, 0.5 mL (25 μg) of fentanyl, and 1 mL of 0.9% sodium chloride. The total volume administered is 2.0 mL. All components were preservative-free and combined in a single syringe under sterile conditions.

After drug administration, patients were continuously monitored for hemodynamic stability. Blood pressure was recorded every two minutes for the first 10 minutes, then at five-minute intervals. Subjective pain intensity was assessed using the Visual Analog Scale (VAS) at four specific time points: prior to the procedure, 10 minutes after administration, one hour after administration, and during delivery.

Data were collected using a structured questionnaire completed 24 hours after delivery to evaluate patient satisfaction and the overall experience with the analgesic technique. The collected responses were analyzed to determine both the effectiveness and acceptability of single-shot spinal analgesia as a potential alternative to epidural analgesia during labor.

Assessment was performed using a structured questionnaire specifically developed for the purposes of this study. Although the instrument had not been previously validated in independent populations, its development was based on expert clinical consensus and current standards in labor analgesia. The English version of the questionnaire is provided in the appendix.

A priori power analysis was conducted using G*Power software (version 3.1) (Heinrich-Heine-Universität Düsseldorf, Düsseldorf, Germany) to determine the appropriate sample size for detecting a minimum clinically significant reduction in VAS score of 2 points, assuming a standard deviation of 2.5, α = 0.05, and power (1−β) = 0.80. The required sample size was estimated at 34 participants. A total of 50 parturients were included to account for potential data exclusion and to enhance statistical robustness.

Descriptive statistics were used to summarize demographic and clinical data. The Wilcoxon signed-rank test was applied to assess changes in VAS scores across time points, while Spearman’s rank correlation coefficient was used to explore associations between parity and both analgesia duration and VAS score. Statistical analyses were conducted using IBM SPSS Statistics, version 26 (IBM Corp., Armonk, NY, US). A p-value < 0.05 was considered statistically significant. The study was approved by the institutional ethics committee and conducted in accordance with the Declaration of Helsinki.

## Results

The analysis was performed on a sample of 50 parturients who received single-shot spinal analgesia during labor. It included both descriptive and inferential statistics, graphical representation of changes in pain intensity, assessment of patient satisfaction, frequency of complications, and correlation of key variables. For numerical variables, mean values, medians, minimum and maximum values, standard deviations, and measures of distribution were calculated.

The average age of the patients was 31 years (range: 22-40), and the mean number of previous deliveries was 1.8 (range: 1-4). On average, labor pain lasted approximately four hours before the administration of analgesia.

**Figure 1:**
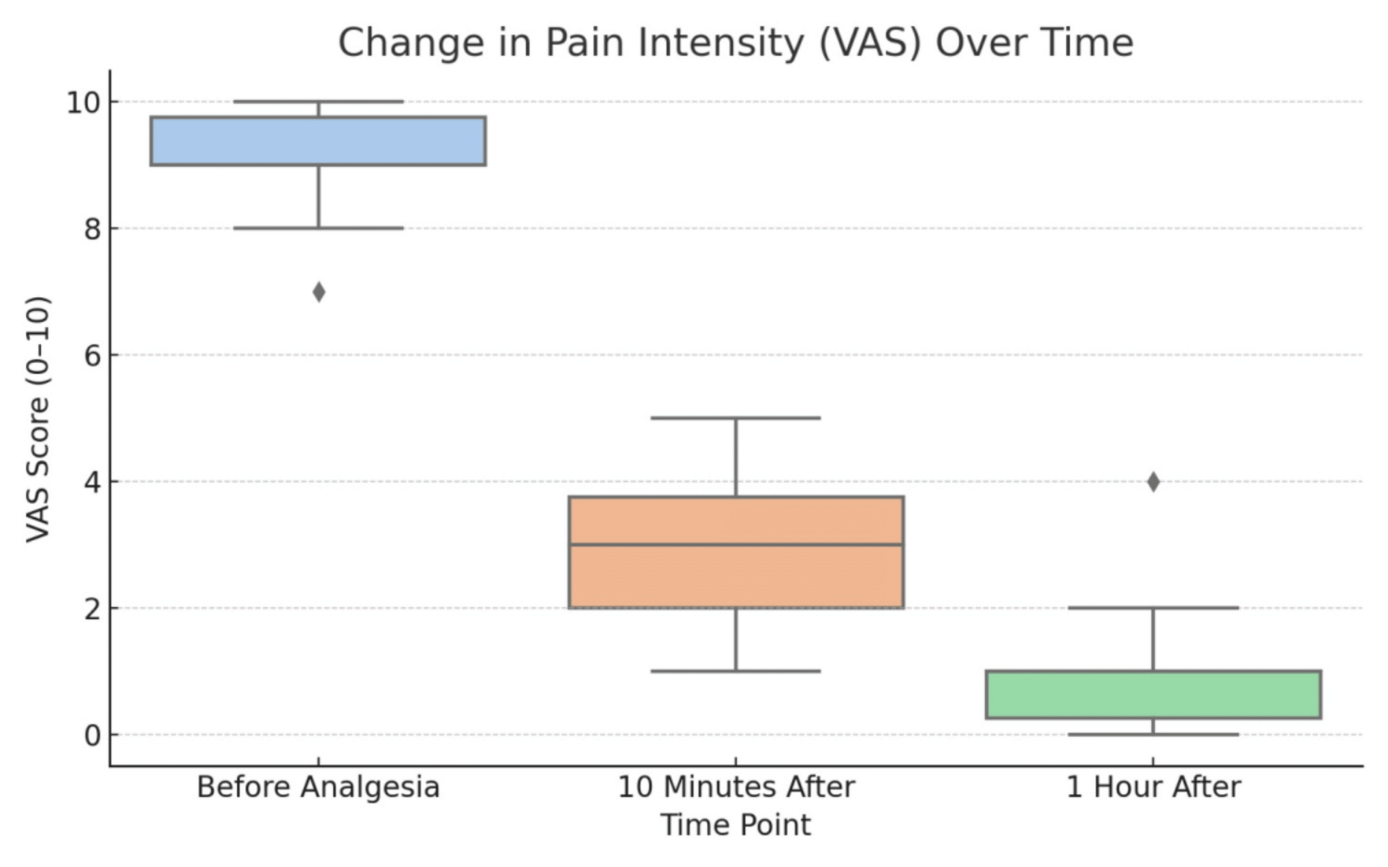
Visual representation of changes in pain intensity at different time points. VAS: Visual Analog Scale

Changes in VAS scores were evaluated using the Wilcoxon signed-rank test for paired samples. The results demonstrated a statistically significant reduction in pain intensity at all measured intervals: before vs. 10 minutes after (p < 0.0000000004), before vs. one hour after (p < 0.0000000004), and 10 minutes after vs. one hour after (p < 0.0000000062). All differences were statistically significant, confirming the high effectiveness of single-shot spinal analgesia. Although we did not include a formal control group, anecdotal reports from the same period suggest that women who did not receive spinal analgesia often continued to experience high VAS scores (typically ≥8) throughout labor, highlighting the added value of this intervention in our setting (Figure 1).

**Figure 2:**
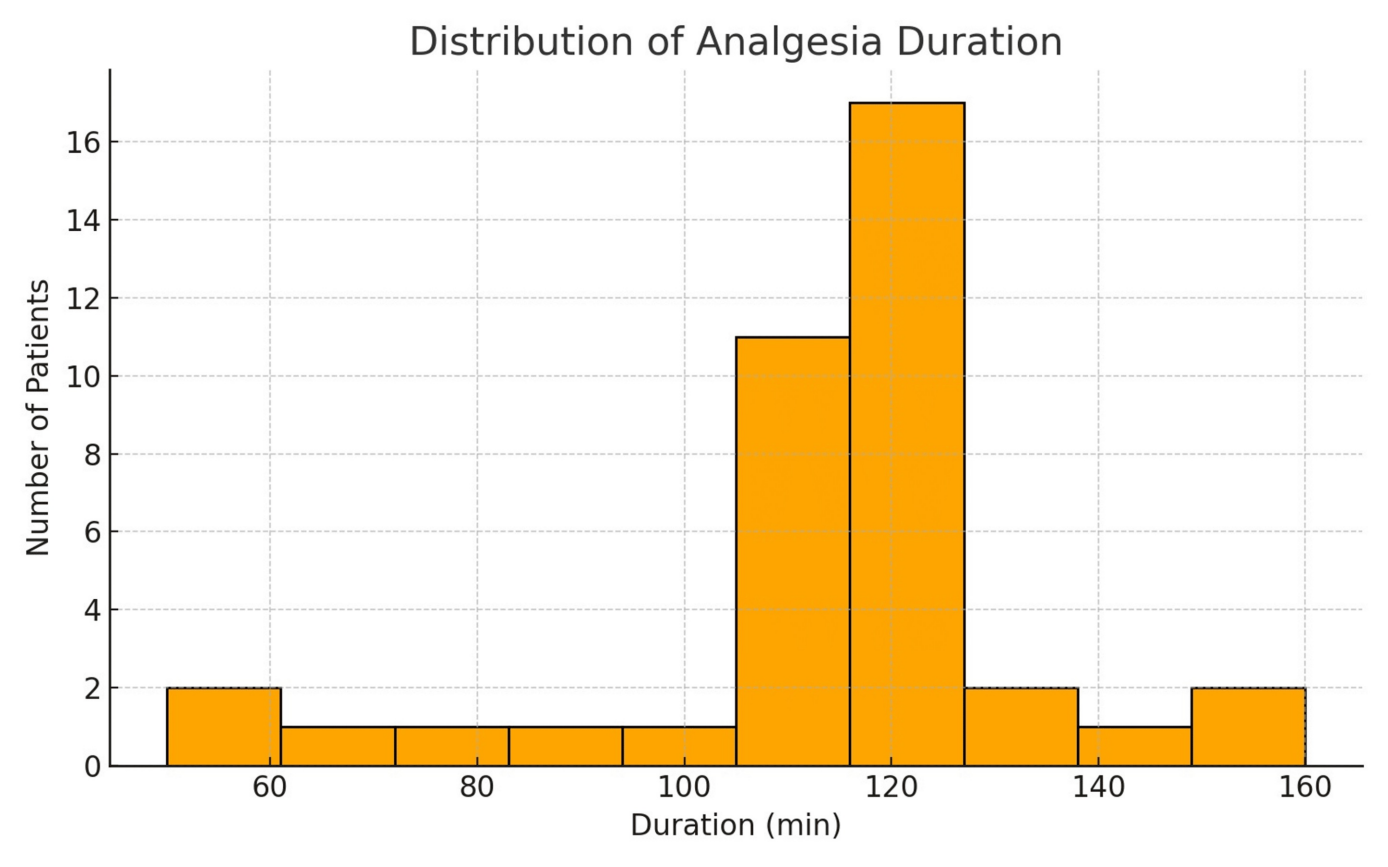
Histogram showing the distribution of analgesia duration among patients. min: minutes

The duration of single-shot spinal analgesia was analyzed in 50 parturients. The average duration was approximately 111.6 minutes, with a median of 110 minutes. Values ranged from a minimum of 50 to a maximum of 170 minutes, with a standard deviation of 18.5 minutes, indicating moderate variability among patients. The distribution of analgesia duration was relatively symmetrical, with most values clustered between 100 and 120 minutes (Figure 2).

This consistency suggests the predictable clinical effectiveness of the technique. These findings are consistent with previous studies showing that single-shot spinal analgesia typically lasts between 1.5 and 2.5 hours (90-150 minutes), which is generally sufficient to cover the active phase of labor in most women. In cases of prolonged labor, additional analgesia or conversion to another technique, such as epidural analgesia, may be considered.

The relationship between the number of previous deliveries and both the duration of analgesia and pain intensity during labor was analyzed. The correlation between parity and analgesia duration was ρ = 0.11 (p = 0.44), while the correlation between parity and VAS scores during labor was ρ = -0.02 (p = 0.88). These analyses were conducted using Spearman’s rank correlation coefficient, as the data were ordinal and not normally distributed. The results were not statistically significant, suggesting that the number of previous deliveries does not meaningfully affect either the duration of analgesia or the subjective experience of labor pain.

All 50 parturients (100%) reported being satisfied with single-shot spinal analgesia during labor. Not a single participant indicated dissatisfaction or partial satisfaction with the method.

Satisfaction was assessed using a structured postpartum questionnaire (Appendix) with three response options: “Satisfied,” “Partially satisfied,” and “Dissatisfied.” All 50 parturients (100%) selected the “Satisfied” option, indicating a uniformly positive response to the analgesic technique.

**Figure 3:**
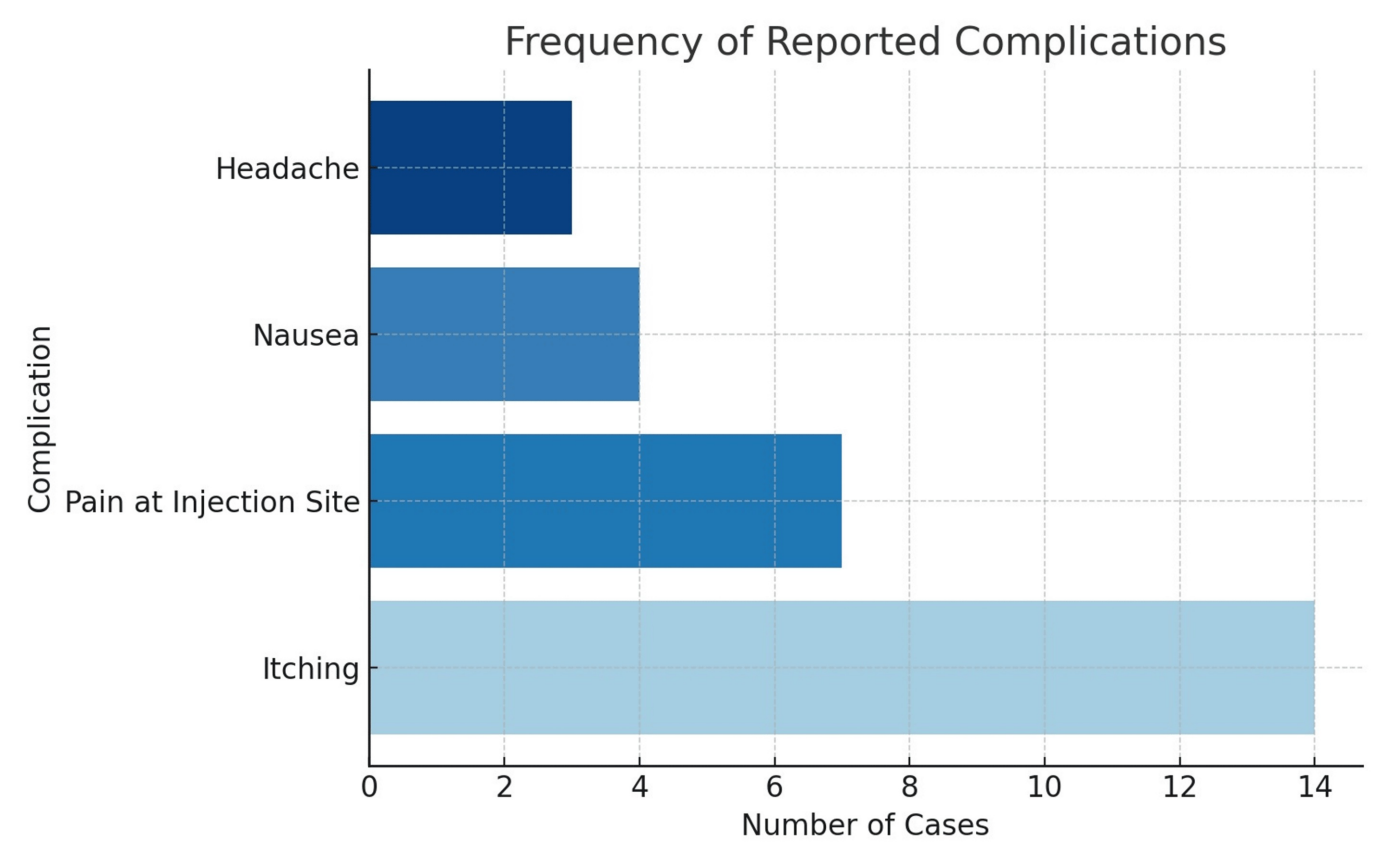
Visual representation of the most commonly reported complications following analgesia administration.

The most commonly reported side effects were itching (28%), pain at the injection site (14%), nausea (8%), and headache (6%). All adverse effects were mild in nature and did not require additional treatment. Importantly, the occurrence of side effects did not negatively impact patient satisfaction. All participants, including those who experienced adverse effects, reported a high level of satisfaction and expressed willingness to choose this method of analgesia again (Figure 3).

Regarding maternal hemodynamics, mild hypotension was observed in 12 patients (24%), defined as a transient decrease in systolic blood pressure of less than 20% from baseline. No cases of severe hypotension were recorded. Only one patient (2%) required vasopressor support, which was managed with a single intravenous bolus of 5 mcg norepinephrine. Importantly, no episodes of bradycardia were observed during the monitoring period.

**Figure 4:**
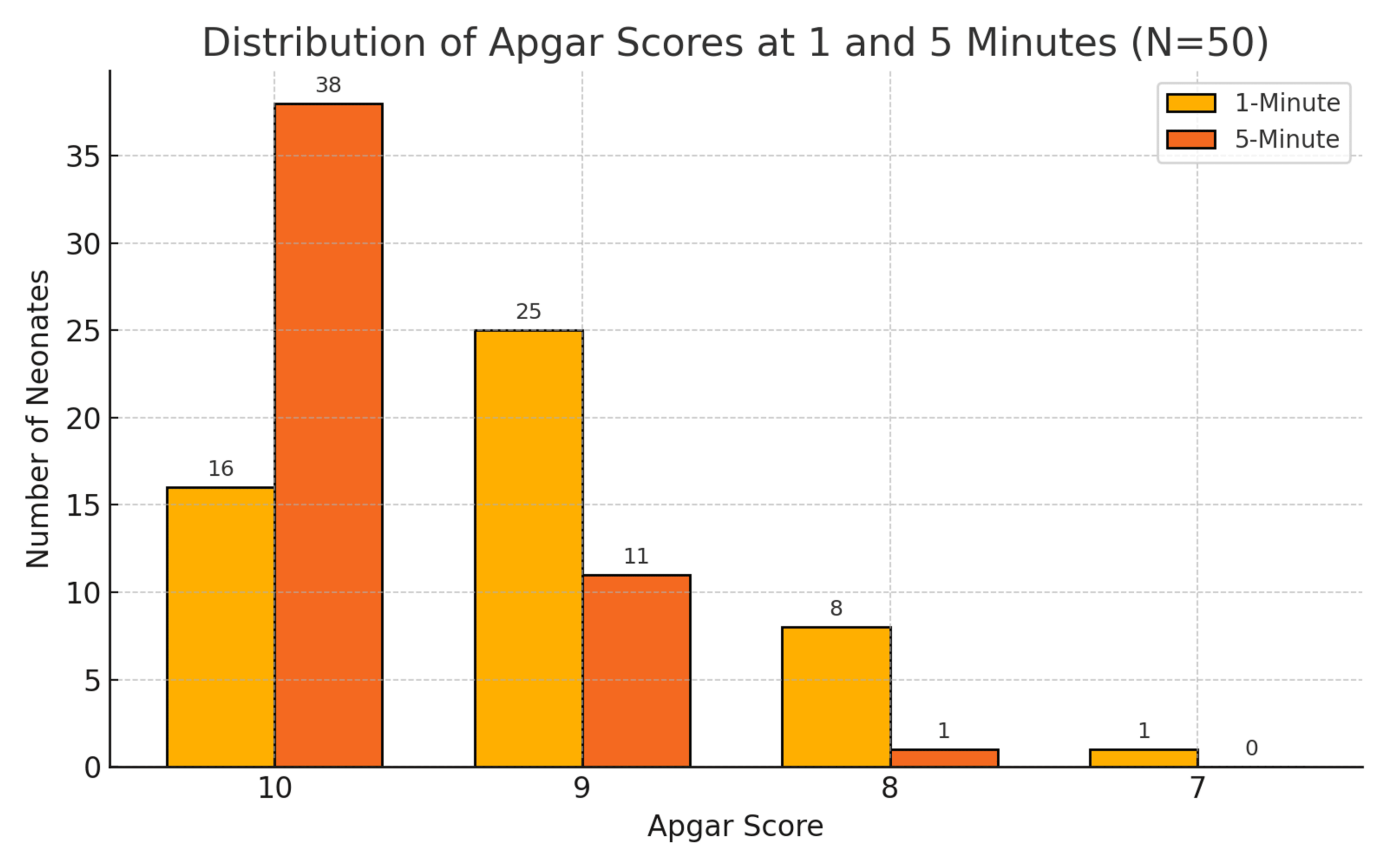
Visual representation of the distribution of Apgar scores at 1 and 5 minutes after delivery. N: number of neonates; Apgar: appearance, pulse, grimace, activity, and respiration

Neonatal Apgar (appearance, pulse, grimace, activity, and respiration) scores were recorded at one and five minutes after delivery. At one minute, 16 neonates (32%) had a score of 10, 25 (50%) scored 9, 8 (16%) scored 8, and one (2%) scored 7. By five minutes, the scores had improved further, with 38 neonates (76%) achieving a score of 10, 11 (22%) scoring 9, and only one (2%) scoring 8. None of the neonates required resuscitation or admission to the neonatal intensive care unit (NICU), indicating favorable neonatal outcomes across the cohort (Figure 4).

The most common source of information regarding the availability of single-shot spinal analgesia was a conversation with the anesthesiologist during labor, typically when the woman requested some form of pain relief. Other sources included obstetricians, other pregnant women, and online forums. This finding highlights a relatively low level of awareness and knowledge among women about the available pain relief options during labor. It underscores the need to improve education and communication between healthcare providers and pregnant women in order to facilitate informed decision-making regarding pain management during childbirth (Table 1).

**Table 1:** Distribution of the source of information about single-shot spinal analgesia among parturients. χ²: chi-squared statistic; %: percentage of patients

Source of information	Number of patients	Percentage (%)	Chi square (χ²)	p-value
Anesthesiologist	38	74.5	35.6	<0.0001
Obstetrician	4	7.8		
Another pregnant woman	4	7.8		
Online forums	4	7.8		

As this was an unblinded study, both patients and clinicians were aware of the intervention, which may have introduced reporting bias in satisfaction ratings. This should be considered when interpreting subjective outcomes.

The results of this analysis indicate that single-shot spinal analgesia is highly effective in providing rapid and significant pain relief during labor, with a high level of patient satisfaction and a low risk of serious complications. These findings support the use of this method as a realistic, safe, and acceptable alternative to epidural analgesia, particularly in resource-limited settings.

## Discussion

The results of this study provide a valuable contribution to the growing body of evidence supporting single-shot spinal analgesia as an effective method for labor pain management. Our primary aim was to assess patient satisfaction with this technique, along with evaluating its effectiveness, duration of action, safety, and overall acceptability. The data obtained indicate that single-shot spinal analgesia resulted in rapid and statistically significant pain relief, with exceptionally high patient satisfaction and a minimal incidence of side effects. These findings are consistent with recent reviews by Rollins et al., which emphasize the expanding role of spinal techniques, especially in centers facing resource constraints or where rapid onset is clinically advantageous [12].

Pain intensity was assessed using the VAS at four time intervals: before administration, 10 minutes after, one hour after, and during labor. The findings revealed an average reduction in pain from an initial score of 9 to 3 after 10 minutes, and down to 1 after one hour. These reductions were statistically significant according to the Wilcoxon signed-rank test (p < 0.0000000004), confirming the clinical effectiveness of the technique. These results are consistent with the findings of Minty et al., which demonstrated that single-dose intrathecal analgesia provides rapid pain relief in laboring women, with minimal side effects [7]. These findings are further supported by Bucklin et al., who demonstrated that intrathecal analgesia provides a significantly faster onset of pain relief compared to epidural techniques, with similarly high maternal satisfaction [13].

Compared to other studies, our findings further confirm that single-shot spinal analgesia can serve as a reliable alternative to epidural analgesia, especially in resource-limited settings. The study by Simmons et al. on combined spinal-epidural techniques indicated that the spinal approach in early labor is more effective for rapid pain control [8]. Our study additionally confirms that although single-shot spinal analgesia is a time-limited method, it provides adequate analgesia for most parturients, with an average duration of 111.6 minutes, which aligns with previously reported ranges (90-150 minutes) [8].

Regarding safety, the analysis of side effects showed that the most frequently reported complication was itching (28%), followed by pain at the injection site (14%), nausea (8%), and headache (6%). All adverse effects were mild and did not require further medical intervention, which is consistent with the findings of Pan and Booth, who reported a similar side effect profile for neuraxial analgesia [2].

The observed satisfaction level was derived from a structured binary-choice questionnaire administered 24 hours after delivery (Appendix). The tool offered three response options (“Satisfied,” “Partially satisfied,” and “Dissatisfied”), with all patients selecting “Satisfied.” Although the instrument was not externally validated, its development was grounded in clinical relevance and patient-centered outcomes. As this was an unblinded study, both patients and clinicians were aware of the intervention, which may have introduced reporting or observer bias. Nonetheless, the uniform positivity likely reflects the significant perceived value of analgesia in a setting where few alternatives are typically available [9].

In our cohort, mild hypotension was observed in 24% of parturients, defined as a transient decrease in systolic blood pressure of less than 20% from baseline. No cases of severe hypotension or bradycardia were recorded. Only one patient (2%) required vasopressor support, which was effectively managed with a single 5 mcg bolus of norepinephrine. These findings are consistent with earlier studies demonstrating that low-dose spinal analgesia provides hemodynamic stability in healthy obstetric populations. Asehnoune et al. showed that small-dose bupivacaine-sufentanil combinations help maintain cardiac output after spinal anesthesia, minimizing the risk of significant hypotension [14]. More recently, Doelakeh and Chandak emphasized that while certain patient- and procedure-related factors may influence the incidence of hemodynamic events during spinal anesthesia, the overall risk in well-selected, low-risk parturients remains low [15].

Interestingly, correlation analysis between the number of previous deliveries and both analgesia duration (ρ = 0.11, p = 0.44) and VAS scores during labor (ρ = -0.02, p = 0.88) showed no statistically significant associations. This suggests that previous childbirth experience does not significantly influence the effectiveness or the subjective perception of spinal analgesia. Clinically, this supports the reliability and consistency of the method regardless of parity.

Our neonatal outcomes further support the safety profile of single-shot spinal analgesia. Apgar scores were uniformly favorable, with 98% of neonates scoring ≥9 at five minutes and none requiring resuscitation or NICU admission. It is important to note that this study was conducted in a secondary-level hospital managing only healthy term pregnancies. Patients with comorbidities or high-risk conditions are typically referred to tertiary centers with specialized obstetric pathology units. This selective patient population likely contributed to the consistently positive neonatal outcomes observed.

These results are in line with existing literature. Ahmadi et al. demonstrated that single-dose spinal analgesia provides rapid pain relief with high maternal satisfaction and no significant neonatal complications [16]. In contrast, Rasmussen et al. found that epidural analgesia in a general obstetric population was associated with higher rates of low Apgar scores and NICU admissions [17]. Taken together, these findings highlight the clinical value of single-shot spinal analgesia, particularly in resource-limited or secondary care settings managing low-risk parturients.

One concerning finding is that 74.5% of the participants learned about the option of spinal analgesia only during labor, mostly through conversations with anesthesiologists. This reflects a generally low level of prior education and awareness regarding pain relief options. It highlights the need to improve prenatal counseling to ensure that women receive timely and comprehensive information about available analgesic techniques. Enhancing education during prenatal visits is essential to enable informed and confident decisions regarding labor pain management. Clear and timely communication about available options, including single-shot spinal analgesia, can significantly enhance the childbirth experience and patient satisfaction. Further advancement in the use of this technique should involve standardizing clinical protocols, ensuring the availability of supplemental analgesia when needed, and developing approaches such as combined spinal-epidural analgesia. In addition, ongoing education of healthcare providers, particularly anesthesiologists, and the establishment of dedicated perinatal anesthesia teams available 24/7 are crucial.

Limitations of this study include the small sample size (50 participants) and the fact that it was conducted at a single institution, which limits external validity. Pain and satisfaction were assessed subjectively, so future studies should aim for larger, multicenter samples and incorporate more objective outcome measures, including neonatal outcomes.

## Conclusions

This study confirms that single-shot spinal analgesia is a safe, effective, and well-accepted method for managing labor pain in healthy term parturients. It offers rapid pain relief, minimal side effects, and high maternal satisfaction, making it a valuable option in environments with limited access to epidural services.

Although single-shot spinal analgesia cannot replace epidural analgesia as the gold standard in modern obstetric care, it serves as a practical and reliable alternative where resources, staffing, or infrastructure are constrained. With appropriate patient education and clear clinical protocols, it can meaningfully enhance the quality of perinatal care, particularly in low-resource settings.

## Appendices

**Table 2:** Patient satisfaction questionnaire administered 24 hours after delivery VAS: Visual Analog Scale Credits: Keser I, Imamović D, Štraus S, and Mihalj M. Questionnaire developed by the authors specifically for this study.

Section	Response options
Year of birth	_________
Number of previous deliveries	_________
Pain intensity before analgesia (VAS 0-10)	_________
Pain intensity 10 minutes after analgesia (VAS 0-10)	_________
Pain intensity one hour after analgesia (VAS 0-10)	_________
Pain intensity during delivery (VAS 0-10)	_________
Satisfaction with spinal analgesia during labor	Satisfied/partially satisfied/dissatisfied
Source of information about regional analgesia	Internet/online forums/another pregnant woman/anesthesiologist/gynecologist/family doctor
Reported complications after spinal analgesia	Pain at injection site/headache/infection at injection site/nausea or vomiting/other: _______/none
Would you choose spinal analgesia again?	Yes/no
Would you recommend spinal analgesia to a friend?	Yes/no
